# Diversity Patterns and Ecological Network Features of Soil Mite Trophic Groups in Karst Cave Ecosystems

**DOI:** 10.1002/ece3.72505

**Published:** 2025-11-20

**Authors:** Yan Shen, Qiang Wei, Yuanyuan Zhou, Ting Song, Yihui Liu, Xiaoxi Lyu, Hua Xiao, Hu Chen

**Affiliations:** ^1^ School of Karst Science Guizhou Normal University Guiyang China; ^2^ Key Laboratory for Information System of Mountainous Area and Protection of Ecological Environment of Guizhou Province Guizhou Normal University Guiyang China

**Keywords:** ecological network, karst caves, Mesostigmata, multidimensional diversity, Oribatida, trophic niche

## Abstract

Trophic niche differentiation alleviates interspecific competition among soil fauna by modulating resource utilization and allocation, offering a novel perspective for understanding biodiversity maintenance and species coexistence in subterranean ecosystems. As a typical example of an oligotrophic and extremely dark environment, karst cave soils support arthropod communities that are functionally distinct from those in surface habitats. However, systematic understanding of the multidimensional diversity patterns and ecological network complexity of multitrophic groups of cave‐dwelling soil mites remains limited. In this study, soil mite samples were collected from distinct photic zones of the cave including the dark, twilight, light, and entrance zones. We aimed to uncover the variation in α, β, and functional diversity among multitrophic groups (including predators and decomposers), and constructed ecological networks to examine patterns of biotic interactions and species coexistence. The results showed that along the cave environmental gradient, the α‐diversity of different trophic groups increased, while functional diversity exhibited a contrasting trend. In addition, except for predator groups, the β‐diversity of soil mites did not differ significantly among cave microhabitats and was primarily driven by species replacement processes. Moreover, the complexity of ecological networks progressively increased along the cave gradient, indicating that interactions among multitrophic groups intensified, with predatory mites playing a central role in maintaining network stability. Notably, no indicator species were found in the dark zone, which represents the core oligotrophic habitat within caves. Our study demonstrates that, compared to surface habitats such as cave entrances and adjacent agricultural lands, cave soil environments support simpler community composition, diversity, and ecological network complexity across different trophic groups of mites. Furthermore, predatory mites not only serve as keystone taxa within cave habitats, but also play a pivotal role in mediating interactions among trophic groups. These findings provide important theoretical insights for understanding community assembly processes under extreme environmental conditions, maintaining ecosystem stability, and supporting the conservation of subterranean biodiversity.

## Graphical abstract

Along karst cave gradients, soil mites show rising α‐diversity yet declining functional diversity toward cave entrances. Predatory mites sustain ecological network stability as keystone taxa despite simplified communities in oligotrophic zones. This reveals biodiversity maintenance mechanisms in extreme subterranean ecosystems.

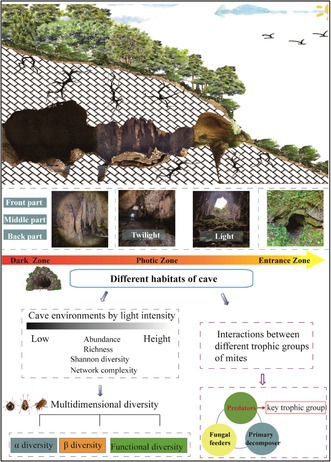

## 1 Introduction

Species diversity and the mechanisms underlying species coexistence have long been central issues in ecological research (Chesson 2000; Nakashizuka 2001; Levine and HilleRisLambers 2009; Potapov et al. 2021). Among these mechanisms, trophic niche differentiation plays a crucial role in maintaining the diversity of soil fauna by alleviating interspecific competition and optimizing resource allocation patterns (Schneider et al. 2004; Schneider and Maraun 2005; Maraun et al. 2011; Klarner et al. 2013; Potapov et al. 2021; Chen et al. 2024; Zhang et al. 2025). Traditionally, soil animals have been regarded as trophic generalists capable of feeding on a wide range of food resources (Scheu 2002a; Digel et al. 2014; Erktan et al. 2020). For example, collembolans and oribatid mites rely on diverse food sources to support their survival and reproduction (Maraun et al. 1998; Scheu and Setälä 2002; Potapov 2022a; Zhang et al. 2023). However, recent studies using stable isotope analyses have revealed that oribatid mites occupy distinct trophic positions, spanning three to six trophic levels (Schneider et al. 2004; Maraun et al. 2011; Klarner et al. 2017; Maraun et al. 2023). These findings have been corroborated by gut content and fatty acid analyses (Heidemann et al. 2011, 2014; Gong et al. 2018; Maraun et al. 2020; Twining et al. 2020). Collectively, these lines of evidence demonstrate pronounced trophic niche differentiation within soil microarthropod communities. Therefore, adopting a research perspective grounded in trophic niche differentiation is essential for deeply understanding the diversity of soil fauna and the ecological mechanisms that sustain it.

Biodiversity plays a fundamental role in maintaining ecosystem functioning and stability (Loreau and Hector 2001; Cardinale et al. 2012). Multidimensional diversity provides a more comprehensive understanding of the mechanisms linking biodiversity to ecological processes (Cadotte et al. 2011). Studies have shown that functional diversity, which integrates species abundance and functional traits, helps elucidate the intrinsic relationships between biodiversity and ecosystem functioning (Gobbi and Fontaneto 2008; Dziock et al. 2011; Simons et al. 2016). For instance, soil microarthropods enhance resource complementarity and key ecosystem processes through diverse feeding strategies and variation in body size (Maaß et al. 2015; Potapov et al. 2022b). In addition, β‐diversity contributes to understanding the spatial structuring of soil animal communities along environmental gradients and their roles in ecosystem functioning. For example, a loss of nestedness due to agricultural intensification can reduce decomposition efficiency, while species turnover along successional gradients may sustain nutrient cycling via functional complementarity (Birkhofer et al. 2015; Widenfalk et al. 2016). Furthermore, species interaction networks offer an integrated framework for examining biodiversity and ecosystem functioning relationships (Thébault and Fontaine 2010; Shao et al. 2016; Wang et al. 2024). In soil ecosystems in particular, trophic interaction networks among distinct functional groups reveal how soil fauna diversity regulates ecological processes through multitrophic cascades (De Vries et al. 2013; Bardgett and Van Der Putten 2014). However, the interaction networks of soil mites and the dynamic relationships among their trophic groups remain poorly understood, limiting our knowledge of how soil biodiversity is maintained and how it contributes to ecosystem functioning.

Karst caves, characterized by oligotrophic conditions and a highly stable environment, serve as natural laboratories for studying the adaptive evolution of subterranean biota (Engel et al. 2010). Based on marked disparities in light intensity, the cave environment is typically divided into the light, twilight, and dark zones (Wilkens et al. 2000). This light gradient, coupled with the microclimatic succession from the entrance toward the deeper sections, collectively shapes a highly heterogeneous cave habitat (Culver and Pipan 2019). Furthermore, cave ecosystems are heavily dependent on external energy inputs such as seasonal flooding, bat guano, and the percolation of surface detritus. The high spatiotemporal uncertainty of these inputs further complicates the distribution of resources (Romero 2009). Soil mites, a group of arthropods highly sensitive to environmental change (George et al. 2017), exhibit community compositions that are closely linked to abiotic factors such as temperature and humidity, as well as the availability of nutritional resources (Wissuwa et al. 2012; Cisneros et al. 2014). In cave ecosystems, the light gradient indirectly establishes the foundational pattern of community differentiation from the entrance to the deeper zones by modulating resource availability. For instance, in the photic zone, the input of surface vegetation litter provides a stable food source for soil mites, typically sustaining higher species diversity (Ren et al. 2021; Prescott 2010). In contrast, community structure in the dark zone is primarily regulated by stochastic external resource inputs. On one hand, allochthonous energy inputs, such as bat guano, can create resource‐rich patches in the deeper cave sections, supporting mite communities of exceptional abundance (Ladle et al. 2012). On the other hand, unfavorable physical conditions, such as persistently high humidity, can exert a strong inhibitory effect on mites (Ganjisaffar and Perring 2015; Döker et al. 2016). Collectively, these phenomena indicate that the combined effects of light and resource inputs govern the distribution patterns and community structure of cave soil mites. Although previous studies have revealed a gradual increase in α‐diversity of soil mites from the dark to the photic zones (Barczyk and Madej 2015; Fei et al. 2024), knowledge remains limited regarding the patterns of multidimensional diversity (such as β‐ and functional diversity), and particularly the trophic structure and ecological network characteristics of their community assembly.

**FIGURE 1:**
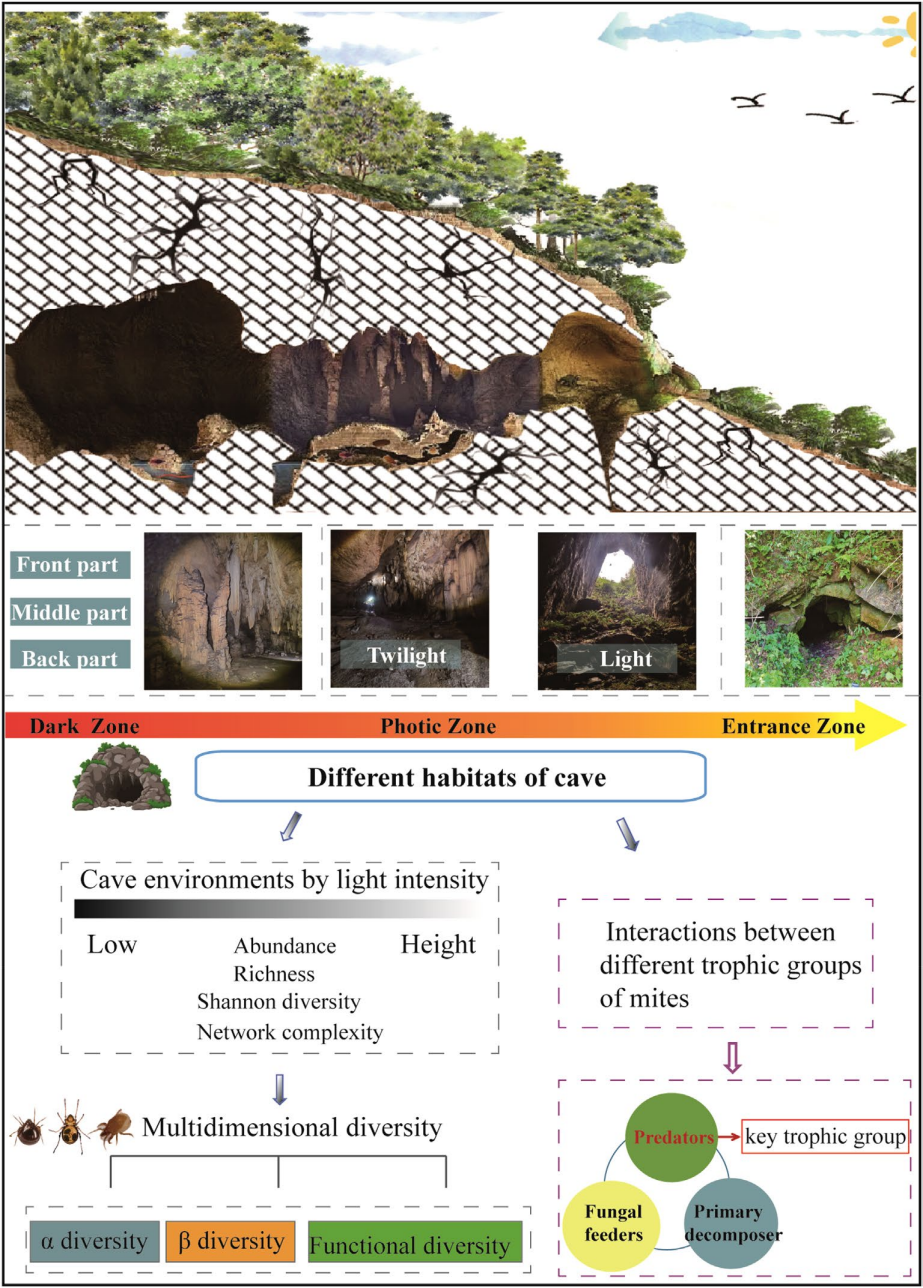
A conceptual model depicting a cave cross‐section and the associated multidimensional diversity and trophic interactions within its soil mite community. The environmental gradient is shown from the interior dark zone, through the photic zone, to the entrance zone. The dark and photic zones are collectively defined as the in‐cave habitat (photographed by Yan Shen, July 2024).

In this study, we investigated soil mite communities in undisturbed natural caves of the karst region in southwestern China. By examining their distribution patterns along a light‐intensity gradient, we aimed to characterize changes in multidimensional diversity and the structural features of ecological networks within cave ecosystems (Figure 1). Building upon prior research on cave‐dwelling faunal communities, we proposed two hypotheses: (1) As light intensity increases from the dark zone to the cave entrance, the α‐diversity and functional diversity of soil mite communities, including those of different trophic groups, are expected to increase. (2) Along the cave environmental gradient, ecological networks will become increasingly complex, with predatory mites—acting as generalist predators—playing a pivotal role in mediating interactions among trophic groups.

## 2 Materials and Methods

### 2.1 Study Site

The Fanjingshan World Heritage Site is surrounded by karst low mountains and hills, creating a transitional zone where karst and non‐karst landscapes intermix. This region harbors rich biodiversity and local endemic species (Guo et al. 2025). The extensive distribution of carbonate rocks here has forged typical karst landforms and a dense cave system on the southeastern side of Fanjingshan (Zhou et al. 2017). The area experiences a subtropical monsoon humid climate with a mean annual temperature of 6°C–17°C and annual precipitation of 1100–2600 mm (Luo et al. 2024). Our study focused on four karst caves located within this transitional zone (27°27′ N–27°58′ N, 108°30′ E–109°06′ E): Longjing Cave, Luzi Cave, Shenjia Cave, and Xiong Cave (Figure S1). Detailed geographical information and basic environmental characteristics for each cave are provided in Tables S1 and S2.

### 2.2 Sample Collection and Identification

In July 2024, we conducted a systematic sampling of caves in the study area. Prior to sampling, we recorded basic information for each cave including geographical coordinates, altitude, and aspect. Subsequently, we progressed from the entrance toward the interior, measuring morphological features such as length, width, and height with a laser rangefinder (Leica D510, Switzerland). To delineate habitat zones, we measured light intensity with a PM6612 illuminometer and classified the cave into three zones: a light zone (> 10 lx), a twilight zone (0.1–10 lx), and a dark zone (< 0.1 lx), following established criteria (Li et al. 1999). In extensive caves, the dark zone was further subdivided into front, middle, and back sections (Figure 1). Within each zone, we established six random sampling points to measure soil temperature and moisture. At each point, three replicate measurements were taken using a TDR200 soil parameter analyzer, and the mean value was used to represent the microclimate for that zone (Table S3). Soil sampling was conducted along a gradient from the deep interior of the cave toward the entrance. Within each designated zone, six soil samples were randomly collected. For comparison, an additional six samples were taken from a nearby agricultural plot (a maize field). In total, 150 samples were collected. Furthermore, in each plot, a composite soil sample was created by collecting and homogenizing soil from multiple points. These samples were then chemically analyzed to determine their total carbon (TC), total nitrogen (TN), total potassium (TK), soil organic matter (SOM) content, and pH. For areas with sufficient soil depth, samples were collected using a stainless‐steel ring (10 cm diameter, 5 cm height). In areas with shallow soil, a volume of soil equivalent to one ring was excavated with a spade. All samples were placed in opaque cloth bags, labeled, and transported to the laboratory for subsequent analysis.

We used a modified Berlese–Tullgren funnel to extract soil fauna in the Soil Zoology Laboratory (Fei et al. 2024). Due to the high moisture content of soil samples collected from cave environments, each sample was continuously heated for 72 h. The extracted fauna were transferred into Petri dishes, and soil mites were manually sorted under a stereomicroscope (Olympus BX53, Japan). Selected mites were then mounted on temporary slides for taxonomic identification under a compound microscope (Olympus CX41RF, Japan). The taxonomic identification of adult soil mites was based on several authoritative references including *A Manual of Acarology* (Krantz and Walter 2009), *Pictorial Keys to Soil Animals of China* (Ying 1998), *Soil Gamasid Mites in Northeast China* (Yin et al. 2013), *Biodiversity of Jilin Province. Fauna: Suborder Oribatida Volume* (Liu 2021), *Oribatid Mites: Biodiversity*, *Taxonomy and Ecology* (Behan‐Pelletier and Lindo 2023), and *Acarology* (Li and Li 1989). All soil mite specimens were identified to the genus level, which has been demonstrated to be sufficient for assessing soil animal diversity and community structure in previous studies (Meehan et al. 2019; Wei et al. 2022; Zhou et al. 2022). All identified specimens were preserved at the School of Karst Science, Guizhou Normal University.

### 2.3 Determination of Functional Traits of Soil Mites

In this study, we quantified four functional traits of soil mites: trophic group, body length, body width, and body size. Mites were classified into four trophic groups: predators, primary decomposers, secondary fungal feeders, and lichen feeders (Schneider et al. 2004; Maraun et al. 2011, 2023; Walter and Proctor 2013; Pan et al. 2023) (Table S4). These groups are defined by their ecological roles: predators consume nematodes, collembolans, and the larvae and eggs of other microarthropods (Koehler 1999; Wissuwa et al. 2012); primary decomposers drive litter fragmentation and stimulate microbial decomposition through feeding and excretion; secondary fungal feeders indirectly influence decomposition by regulating microbial communities through fungal consumption; and lichen feeders specialize in consuming lichens, contributing to the breakdown of organic material on cave surfaces (Wissuwa et al. 2012; Magilton et al. 2019; Maraun et al. 2011; Pan et al. 2023). To ensure the validity of our analyses, we excluded the lichen‐feeder group, as it was represented by only a single genus (*Camisia*) in our samples. Mite body length and width were measured directly using a VHX‐5000 digital microscope. For each sample, all individuals of a given genus were measured if fewer than 15 were present; otherwise, 15–20 individuals were randomly selected for measurement. Finally, mite body size was represented by fresh body mass, which was calculated from the measured length and width using a power‐law equation (Newton and Proctor 2013; Potapov et al. 2019).

(1) $$\mathrm{FM}=2.14\times 10^{-7}\times L^{1.53}\times W^{1.53}$$

In the equation, FM represents fresh body mass (μg), *L* is body length (μm), and *W* is body width (μm). The mean value for each taxonomic group was then calculated from the measured individuals.

### 2.4 Statistical Analysis

All statistical analyses were conducted using R version 4.0.3 (R Core Team 2020), with data visualizations generated via the “ggplot2” package (version 3.5.0; Wickham 2016). Ecological networks were visualized using Gephi software (version 0.9.2). Abundance data at each sampling site were standardized to individual counts per square meter (1 m^2^) to represent population density. Given the high number of empty samples (*n* = 27) in the dark zone of the caves, the front, middle, and back sections were merged and treated as a single dark zone. Additionally, due to the short length of the twilight and photic zones, these sections were combined and categorized as the cave photic zone. The cave entrance zone was defined as the transitional habitat at the cave mouth (Figure 1), while adjacent farmland was used as an external reference habitat. To visualize the environmental gradient within caves, we compiled environmental data from 16 caves across a light gradient, combining our findings with those from previously published studies (Figure S2).

We calculated the alpha diversity of the mite community, including richness, Shannon diversity index, and Pielou's evenness index, using the “vegan” package (version 2.6‐2; Oksanen et al. 2022). To test for differences among habitats, we performed a one‐way analysis of variance (ANOVA). Before analysis, the assumptions of normality and homogeneity of variance were checked using the ‘performance’ package. When the ANOVA results were significant (*p* < 0.05), Fisher's Least Significant Difference (LSD) post hoc test, implemented in the “agricolae” package, was used to identify specific pairwise differences. For all statistical tests, the significance threshold (*α*) was set at 0.05. To visualize the community composition, we generated a stacked bar chart showing the relative abundance of the top ten mite taxa across the different cave habitats. We then used the “UpSetR” package (v1.4.0; Gehlenborg 2019) to create an UpSet plot, which illustrates the number of taxa that are unique to a single habitat, shared by multiple habitats, or common to all four habitats. To identify identified indicator genera for each habitat by calculating Indicator Values (IndVal) at the genus level using the “indicspecies” package (v2.0.3; De Cáceres and Legendre 2009). This metric assesses both the fidelity and abundance of a genus within a particular habitat. Genera with an IndVal score exceeding 70% were considered significant indicators of that habitat (Botes et al. 2006).

To assess differences in mite community and trophic group composition among habitats, we performed non‐metric multidimensional scaling (NMDS) based on Bray‐Curtis dissimilarities using the “vegan” package (v2.6‐6; Oksanen et al. 2022). The statistical significance of these differences was then tested with an Analysis of Similarities (ANOSIM) based on 999 permutations. Beta diversity was subsequently partitioned using the beta.div.comp function in the “adespatial” package (v0.3‐8; Dray et al. 2020) to quantify the relative contributions of species turnover and richness differences. To analyze functional diversity, we selected four key functional traits: trophic group, body length, body width, and body size. Functional diversity indices and community‐weighted means (CWM) of body shape were then calculated using the dbFD function in the “FD” package (v1.0‐12.3; Laliberté and Legendre 2010) and the cwm function in the “BAT” package (v2.9.6; Cardoso et al. 2024), respectively.

To examine the interactions among mite communities across different habitats and the trophic interactions within cave environments, we constructed co‐occurrence networks for each habitat based on Spearman's rank correlation matrices using the “igraph” package (Csardi and Nepusz 2006). We also built an integrated co‐occurrence network encompassing all trophic groups within cave habitats, from which we extracted subnetworks to investigate both inter‐ and intra‐group trophic interactions. The topological properties of each network were calculated using “igraph” to evaluate network complexity across habitats and to quantify structural complexity among different trophic groups. To identify key taxa within these networks, we performed node modularity analysis using the “microeco” package (Liu et al. 2021). Nodes were classified based on their within‐module degree (*Z*_*i*_) and among‐module connectivity (*P*_*i*_), following the criteria proposed by Olesen et al. (2007): connector nodes (*Z*_*i*_ < 2.5, *P*_*i*_ ≥ 0.62) and peripheral nodes (*Z*_*i*_ < 2.5, *P*_*i*_ < 0.62). Mite taxa identified as connector nodes were considered keystone groups within the network.

## 3 Results

### 3.1 α‐Diversity of Mite Communities and Trophic Groups

**FIGURE 2:**
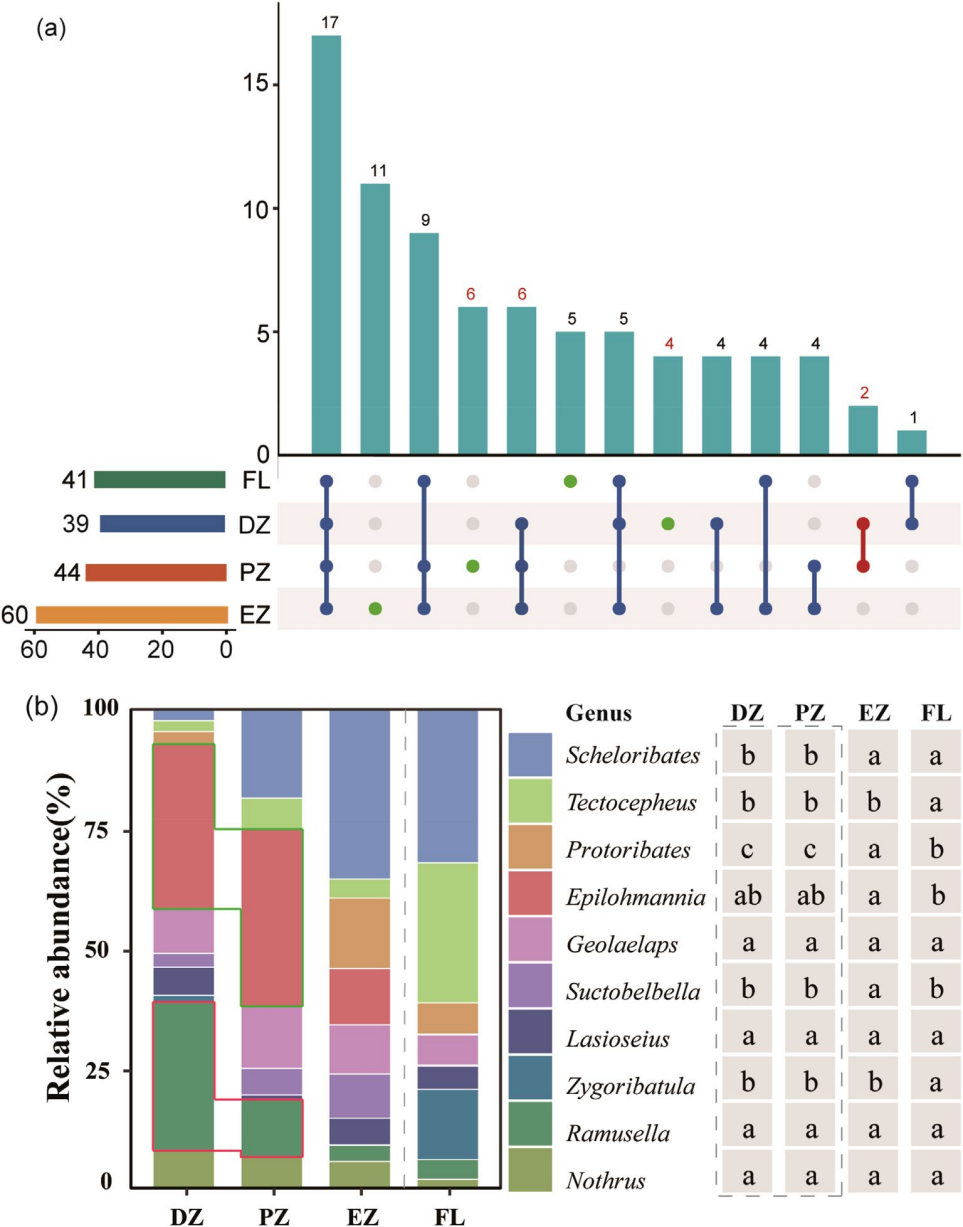
Community composition patterns of soil mites in the cave ecosystem. (a) UpSet plot showing genus‐level composition across habitats. The top bar plot indicates the number of unique genera for each habitat combination; the left bar plot shows the total number of genera observed in each habitat; the right dot matrix illustrates unique genera (single green dots) and shared genera (dots connected by blue lines). (b) Distribution of dominant mite genera (top ten in relative abundance). Bar plots show the relative abundance of each genus in the four habitats. The table on the right summarizes the statistical differences in relative abundance among habitats. Different letters indicate significant differences (*p* < 0.05). DZ, dark zone; EZ, entrance zone; FL, farmland; PZ, photic zone.

We identified 2718 mites from 78 genera and 46 families, spanning the Mesostigmata and Oribatida orders (Table S5). The trophic groups comprised 36 predators, 33 fungal feeders, 8 decomposer, and one lichen fedder (Table S4). The composition of the top ten most abundant genera shifted across habitats. Specifically, *Epilohmannia* and *Ramusella* were the dominant genera in the dark and photic zones of the cave, respectively, whereas *Scheloribates* and *Tectocepheus* dominated the cave entrance and agricultural land (Figure 2b). Notably, the dominant genera within the cave habitats were all fungal feeders. Although the top ten genera did not differ significantly between the cave's dark and light zones, three genera (*Scheloribates*, *Protoribates*, and *Suctobelbella*) were significantly more abundant in the interior cave zones than in the entrance (Figure 2b; Table S6).

Within cave environments, 4, 6, and 11 unique genera were identified in the dark zone, photic zone, and entrance zone, respectively. Two genera were shared between the dark and photic zones. All unique and shared genera within the cave habitats belonged to either predatory or fungivorous mites (Figure 2a), with predatory mites being predominant. Notably, no indicator genera were detected in the dark zone. In contrast, *Sphaerochthonius* and *Microppia* were identified as indicator genera in the photic zone, while nine genera were recognized as indicator taxa in the entrance zone (Table S7).

**FIGURE 3:**
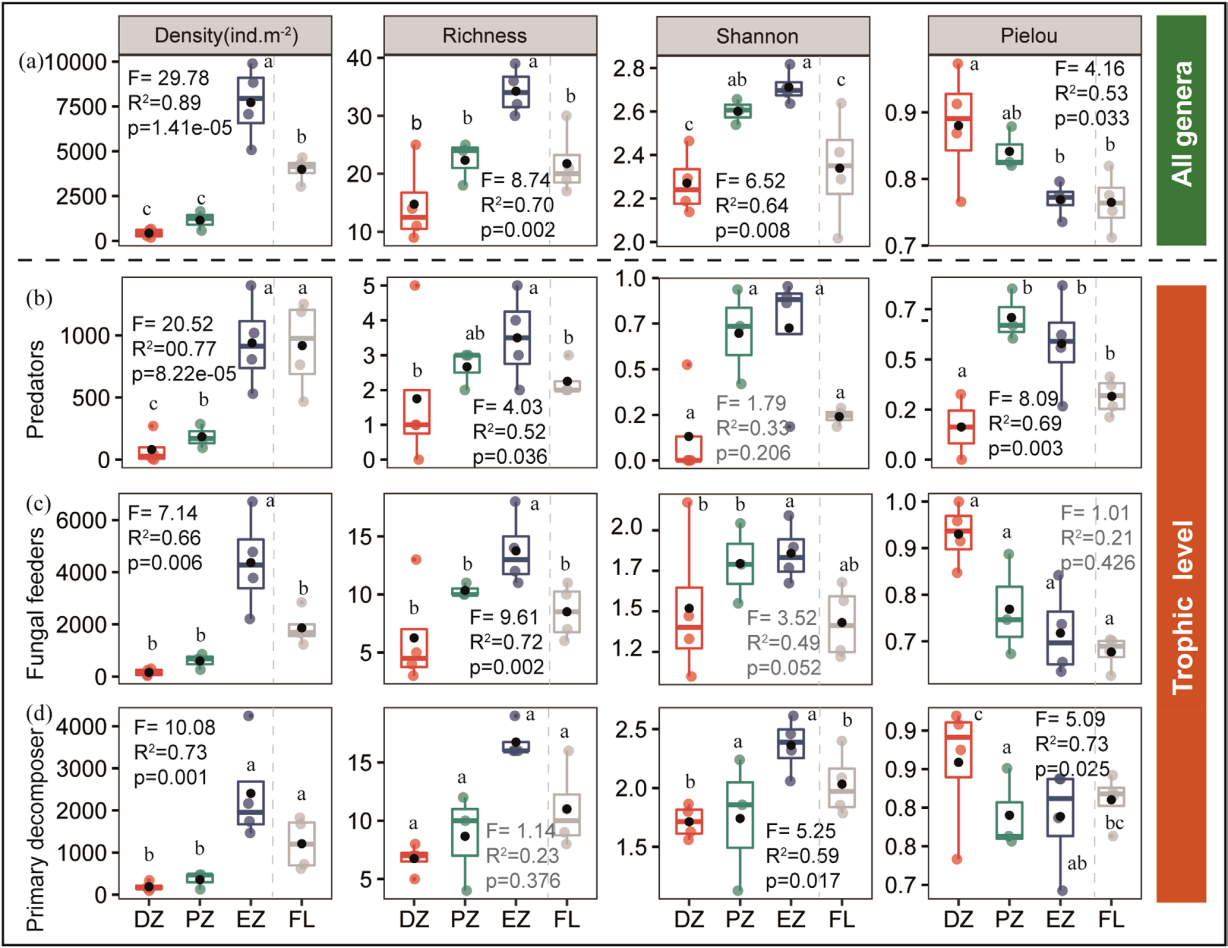
Density and α‐diversity of soil mite communities in different habitats. Box plots show soil mite density, Shannon–Wiener index, Simpson index, and species richness across the four habitats. Results from the one‐way ANOVA (*F*‐statistic, *R* ^2^, and *p*‐value) are indicated. Different lowercase letters above the boxes denote significant differences among habitats. DZ, dark zone; EZ, entrance zone; FL, farmland; PZ, photic zone.

Along the gradient from the cave dark zone to the entrance zone, the density and α‐diversity indices (richness and Shannon diversity) of soil mite communities and their trophic groups showed an overall increasing trend, peaking in the entrance zone (Figure 3). Specifically, the abundance of mites and their functional groups in the entrance zone was significantly higher than in the dark and photic zones of the caves—approximately four times greater than the values observed within cave habitats. Within the cave habitats, only predatory mites exhibited a significant difference in abundance between the dark and photic zones. Richness of both mite communities and trophic groups showed no significant differences among the cave habitats or between the cave habitats and disturbed farmland, but was significantly higher in the entrance zone, approximately double the values recorded in the cave zones. In contrast, Shannon diversity and evenness displayed opposite patterns across habitats, and among the trophic groups, only predatory mites exhibited no significant differences across any of the habitats.

### 3.2 Compositional Differences in Mite Communities and Trophic Groups

**FIGURE 4:**
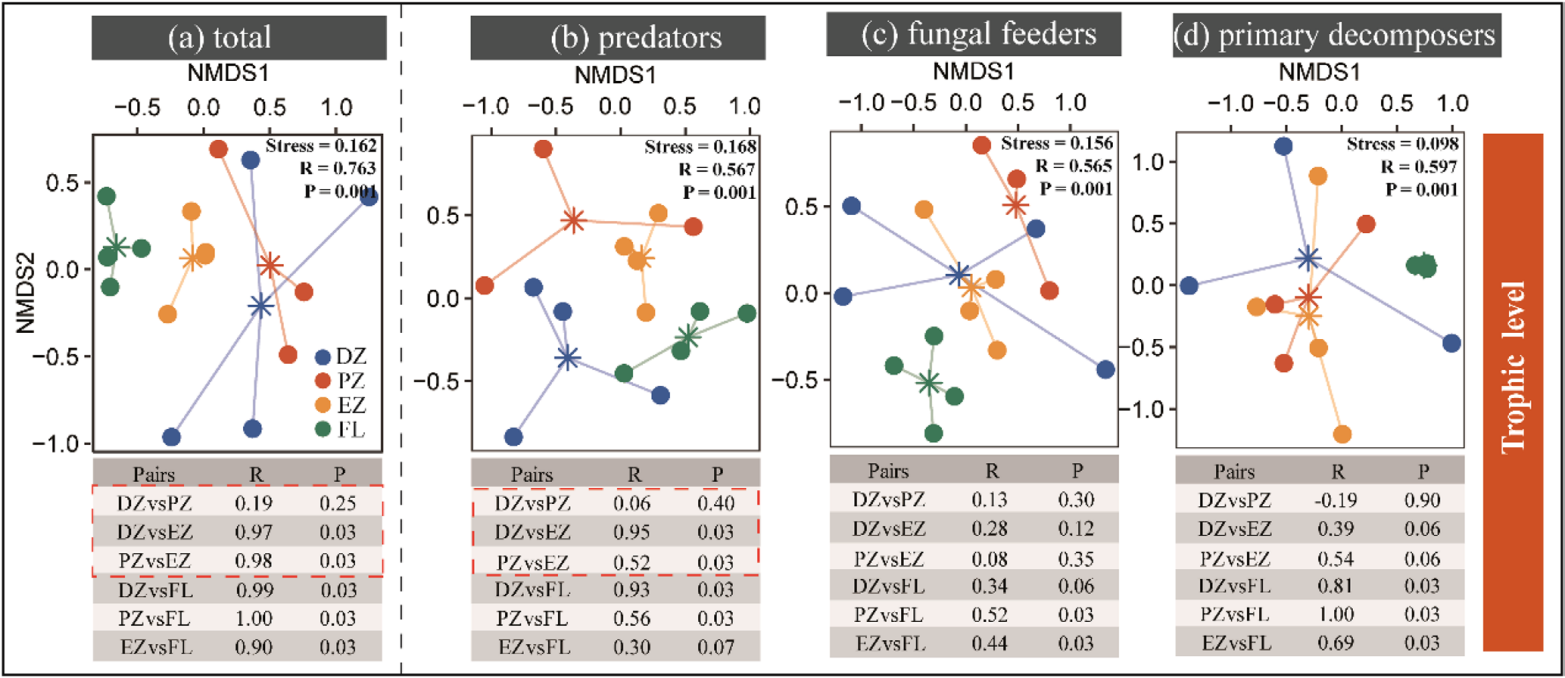
Non‐metric multidimensional scaling (NMDS) plots based on Bray–Curtis dissimilarities. Plots show the composition of (a) the overall mite community and (b–d) trophic groups across habitats. Significant differences in community composition were tested using analysis of similarities (ANOSIM) with 999 permutations. DZ, dark zone; EZ, entrance zone; FL, farmland; PZ, photic zone.

**FIGURE 5:**
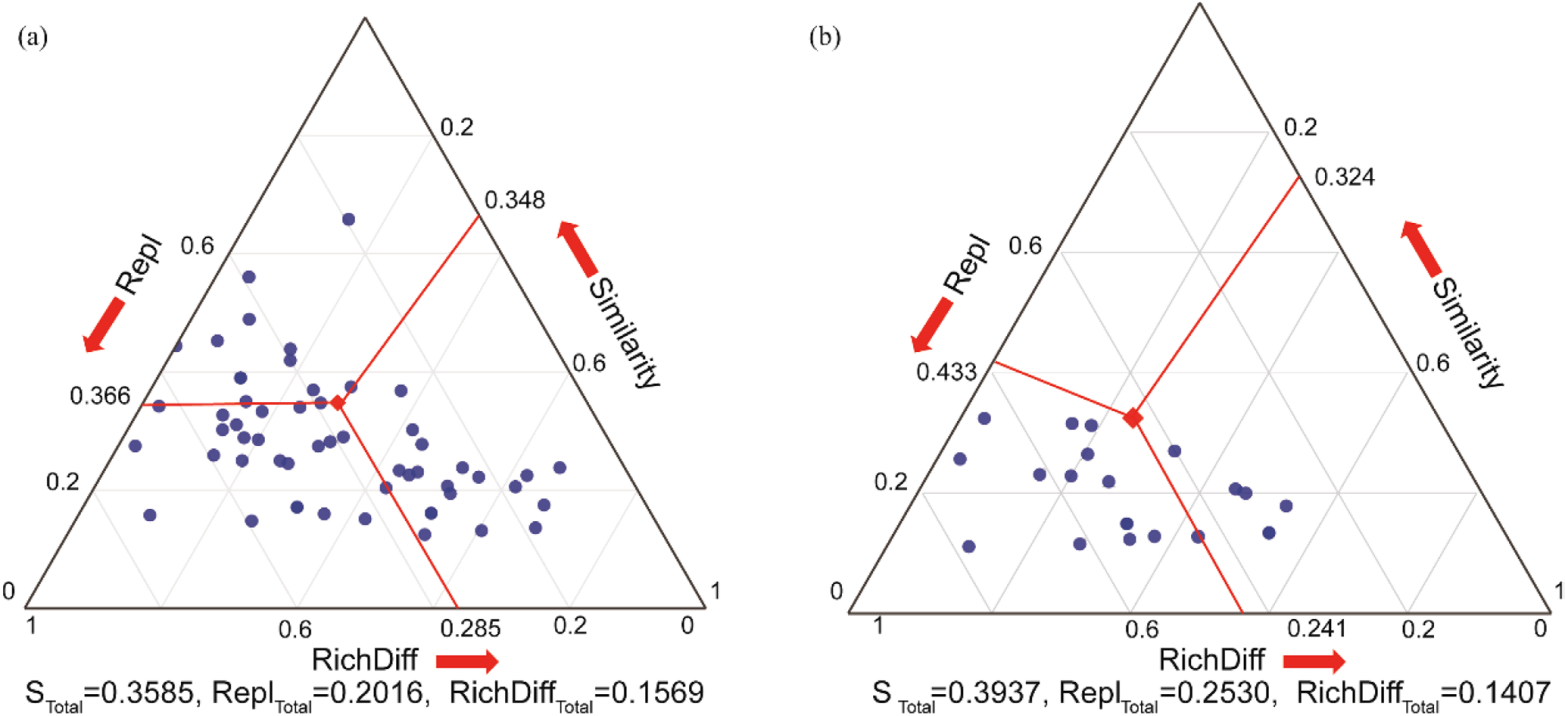
Triangular plots illustrating β‐diversity components of soil mite communities: (a) comparisons across all sampling sites in the cave and entrance zone; (b) comparisons within cave habitats. Analyses were based on the Sørensen dissimilarity index. Each point represents a pairwise comparison between two sampling sites, positioned according to the triplet values of similarity (S), species replacement (Repl), and richness difference (RichDiff), with their sum equal to 1. The large central dot in each plot represents the centroid of all pairwise comparisons, while lines extending toward each axis indicate the group‐wise mean values. Repl and RichDiff reflect the respective contributions of species turnover and richness difference to overall β‐diversity.

Non‐metric multidimensional scaling (NMDS) revealed significant habitat‐associated heterogeneity in the composition of soil mite communities and their trophic groups (Figure 4). Overall, there were no significant differences in community composition between the dark and photic zones within cave habitats. However, within the cave environment, the composition of predatory mites differed significantly between the entrance zone and both the dark and photic zones. Additionally, both the overall mite community and its trophic groups showed significant compositional differences between cave habitats and farmland. At the scale of the entire cave system, species turnover was the dominant component of β‐diversity, accounting for 56.23%, while variation in species richness contributed 43.76%. Similarly, β‐diversity across the cave zones (dark, photic, and entrance) was also primarily driven by species turnover processes (Figure 5; Table S8).

### 3.3 Effects of Cave Habitat Types on Functional Diversity and Trait Composition of Mite Communities

**FIGURE 6:**
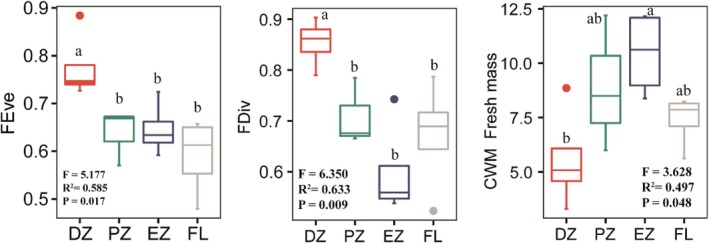
Functional diversity (FD) and community‐weighted mean (CWM) body length of soil mite communities in different habitats. Box plots show FD and CWM body length across the four habitats. Results from the one‐way ANOVA (*F*‐statistic, *R* ^2^, and *p*‐value) are indicated. Different lowercase letters above the boxes denote significant differences among habitats. DZ, dark zone; EZ, entrance zone; FL, farmland; PZ, photic zone.

Functional diversity indices of soil mite communities exhibited a declining trend along the cave environmental gradient. Functional evenness (FEve) and functional divergence (FDiv) were highest in the dark zone, showing significant differences from all other habitats. In contrast, the community‐weighted mean (CWM) of body size (as fresh mass) followed an opposite pattern, peaking in the entrance zone. Moreover, the CWM of fresh mass in the dark zone was significantly lower than in all other habitats (Figure 6; Table S4).

### 3.4 Co‐Occurrence Networks of Mite Communities and Trophic Groups

**FIGURE 7:**
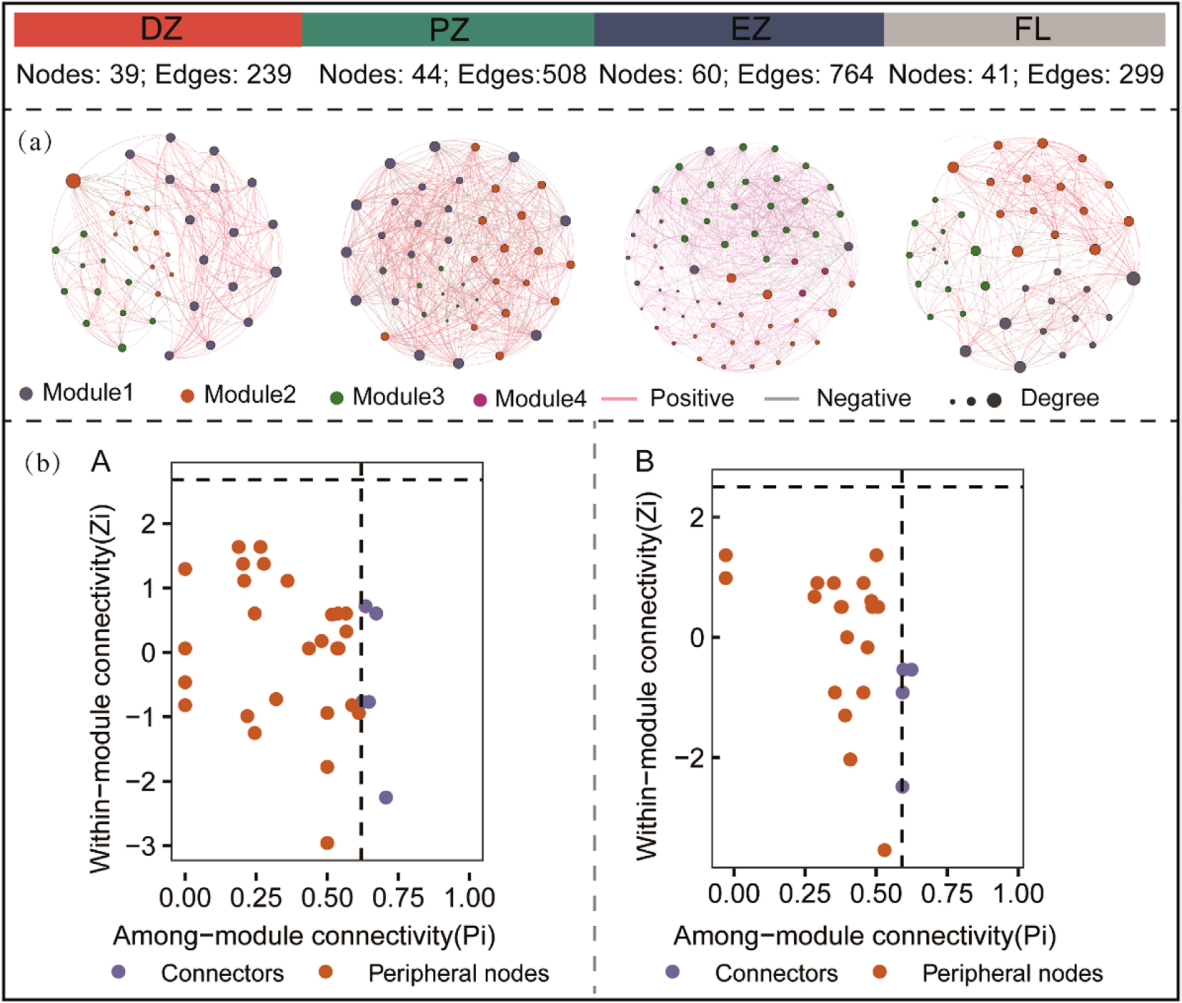
Network analysis of mite communities. (a) Co‐occurrence networks for mite communities in different habitats. From left to right: The dark zone, photic zone, entrance zone of the cave, and farmland. Node size is proportional to the number of connections (degree). Nodes are colored according to their taxonomic classification and module membership. Pink and gray edges represent positive and negative correlations, respectively. (b) Intra‐modular (*Z*_*i*_) and inter‐modular (*P*_*i*_) connectivity scores of nodes within the co‐occurrence networks for the A cave interior and B entrance environments. Nodes are classified as peripherals (*Z*_*i*_ < 2.5, *P*_*i*_ < 0.62) or connectors (*Z*_*i*_ < 2.5, *P*_*i*_ > 0.62). DZ, dark zone; EZ, entrance zone; FL, farmland; PZ, photic zone.

Soil mite communities in different habitats formed co‐occurrence networks, each exhibiting distinct topological properties. Along the cave environmental gradient from the dark zone to the entrance zone, the number of nodes, edges, average degree, and negative edges in the networks showed an increasing trend, reaching their highest values in the entrance zone (dark zone < photic zone < entrance zone). The network in the entrance zone exhibited a higher average degree, indicating more complex species interactions, while the network in the dark zone displayed the highest clustering coefficient and modularity, suggesting more cohesive but compartmentalized structures. Among all habitats, the farmland—subject to anthropogenic disturbance—ranked second to the dark zone in terms of nodes, edges, negative edges, and clustering coefficient, while its modularity value was the highest among the four habitats (Figure 7a; Table S10). Finally, regarding the cave's interior and exterior environments, 12 and 6 genera in the soil mite interaction networks of the interior and entrance habitats, respectively, met the criteria of *Z*_*i*_ < 2.5 and *P*_*i*_ ≥ 0.62. Therefore, these 18 genera in total constituted the keystone taxa of the network. Further analysis revealed that the keystone taxa within the interior cave habitats were predominantly predatory mites (Figure 7b; Table S9).

**FIGURE 8:**
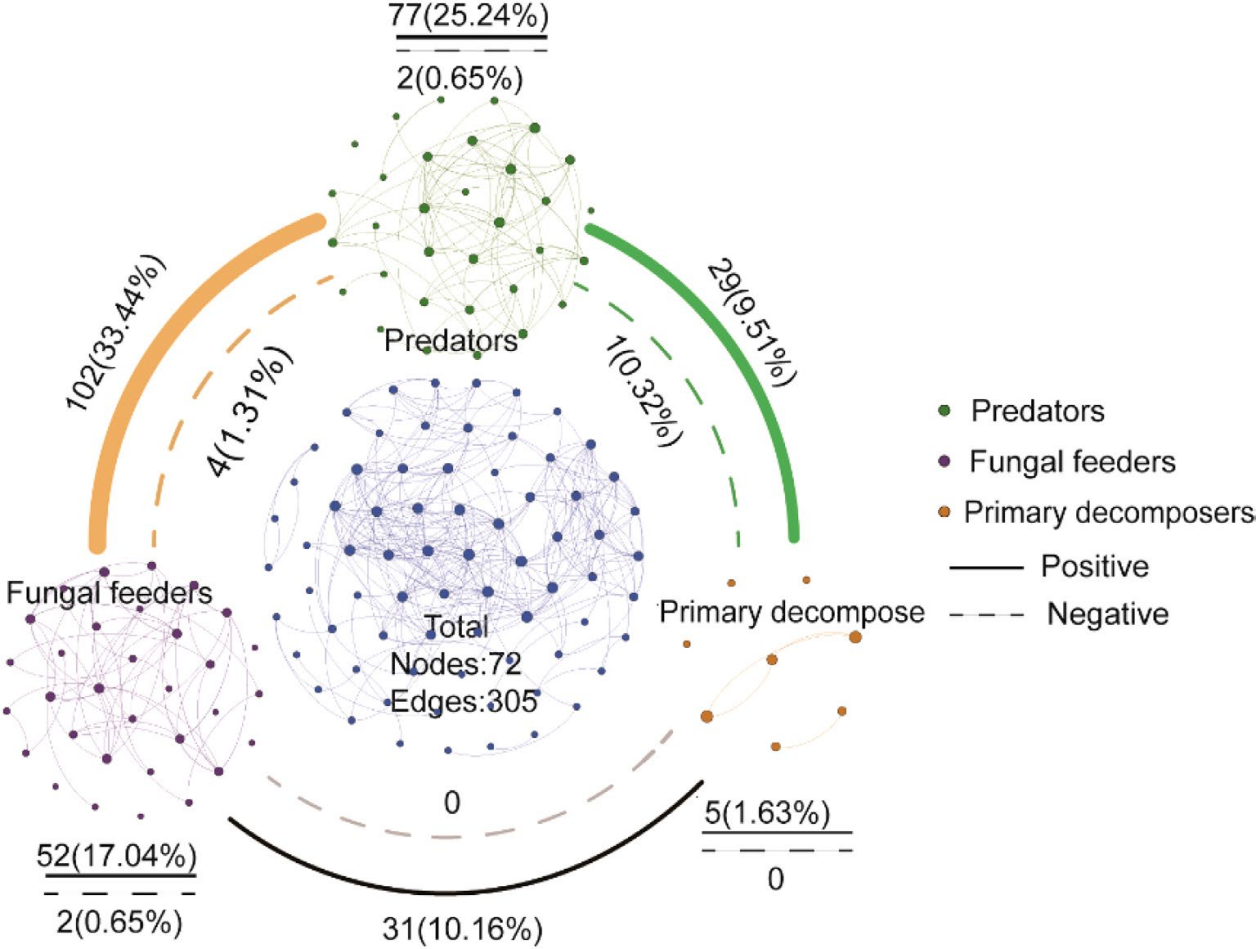
Co‐occurrence network of multitrophic soil mites from inside and outside the cave. The network illustrates co‐occurrence patterns of the soil mite community, structured by three trophic groups. Nodes represent genera, and edges represent inter‐generic associations: Solid lines indicate positive correlations, while dashed lines indicate negative correlations. Edge colors distinguish association types, with black for intra‐trophic and colored for inter‐trophic connections. Edge width is proportional to the prevalence of that association type, and the frequency value is labeled adjacent to the edge.

In cave habitats, trophic interactions within the mite community formed multitrophic co‐occurrence networks. Specifically, positive correlations dominated both within and between trophic groups. Notably, among the extracted subnetworks, the network formed by predatory mites accounted for the highest proportion of links (25.24%, 79 edges). Cross‐trophic group analysis further revealed that predatory mites formed strong trophic links with secondary fungivores and primary decomposers, whereas trophic links between the latter two groups were relatively weak, with no negative interactions observed (Figure 8; Table S11).

## 4 Discussion

### 4.1 Multidimensional Diversity of Soil Mite Communities and Trophic Groups Across Cave Habitats

Our results reveal a significant increase in mite density, richness, and Shannon diversity along the environmental gradient from the dark zone to the entrance of the cave, a pattern consistent with previous studies (Ducarme et al. 2004; Simões et al. 2015; Barczyk and Madej 2015; Fei et al. 2024). We further demonstrate that different trophic groups of mites exhibit a similar response. In contrast to these trends in α‐diversity, however, functional diversity showed no significant gradient, contrary to our first hypothesis. This discrepancy suggests that the ecological drivers governing the abundance and α‐diversity of mite communities and their trophic groups are distinct from those influencing functional diversity. The primary driver for the observed decrease in mite density and α‐diversity from the entrance to the dark zone is the gradual attenuation of resources such as light and organic matter input (Barczyk and Madej 2015; Fei et al. 2024). This pattern, where resource‐poor conditions support fewer species, is consistent with classic niche theory (Cardinale et al. 2012). For instance, increased light availability near the entrance promotes plant diversity, which in turn enhances soil organic matter via litterfall, providing a richer food resource for mites (Ren et al. 2021; Prescott 2010). Further investigation into cave ecosystems has revealed that mite community composition is significantly shaped by temperature and moisture, correlating positively with the former and organic matter but negatively with water content (Fei et al. 2024). This negative correlation is particularly pronounced in the dark zone, suggesting that excessive moisture may constrain the distribution of mites in these habitats. On the other hand, the highly stable yet resource‐poor environment within the cave, particularly in the dark zone, acts as a powerful ecological filter, selecting for species with highly specialized functional traits (Protas and Jeffery 2012). This process can drive functional convergence, wherein despite a loss of species, the remaining community adopts similar survival strategies such as starvation resistance and efficient foraging. This convergence effect counteracts the functional dissimilarity caused by species loss, ultimately maintaining functional diversity at a broader scale (Keddy 1992). We propose that this functional convergence stems from the differential utilization of resource gradients by various trophic groups. In the resource‐poor dark zone, for example, the abundance of primary decomposers is directly limited by organic matter input. Fungivores, in contrast, depend on microbial biomass, whose response to environmental change is more complex and not strictly linear. Predatory mites are influenced by a combination of prey availability and microclimate such as humidity. It is this differential response and utilization of resource heterogeneity across trophic levels that maintains the community's overall functional trait space, even as species composition changes, thereby explaining why functional diversity remains stable along the gradient, unlike α‐diversity.

Within the cave's interior, fungivorous and predatory mites are not only endemic assemblages but also serve as indicator groups for the entrance zone. This phenomenon is closely linked to the unique resource conditions of this area. Compared to the resource‐depleted dark zone, the photic zone supports higher primary productivity, fostering a more complex food web (Culver and Pipan 2019; Mammola et al. 2018). This provides a continuous and diverse prey base for predatory mites such as nematodes and collembolans. As generalist predators, their ecological strategy is highly coupled with the high resource heterogeneity of the photic zone, enabling them to flexibly exploit diverse resources and occupy a dominant niche in this patchy environment (McMurtry and Croft 1997; Kondoh 2003; Jensen et al. 2019). For instance, their predation on various lower‐trophic‐level organisms exemplifies their efficient utilization of the habitat's abundant resources (Koehler 1999; Wissuwa et al. 2012). This advantage, however, is absent in the environmentally homogeneous and resource‐poor dark zone. Therefore, the dependence of predatory mites on the specific resource structure of the photic zone is likely the key reason for their status as indicator groups in this region. Furthermore, our study reveals that the pattern of β‐diversity turnover for predatory mites differs from that of other groups. We attribute this to a divergence in community assembly mechanisms. The dominance of the species turnover component in β‐diversity suggests that environmental filtering—driven by factors such as organic matter, temperature, and humidity—is a key driver of community differentiation. For predatory mites, however, their distribution appears to be more strongly driven by the spatial heterogeneity of their prey communities, rather than solely by abiotic gradients. This reliance on biotic patterns results in their distinct β‐diversity signature.

Moreover, by integrating species' functional traits with their relative abundances, functional diversity provides a powerful measure of shifts in community functional composition (Mason and Mouillot 2013; Maaß et al. 2015). Along the cave environmental gradient, both functional divergence (FDiv) and functional evenness (FEve) declined, with peak values observed in the dark zone. In this low‐light environment, consistent environmental stress appeared to promote a more balanced distribution of traits across species, yielding elevated FEve values (Villeger et al. 2008). This pattern suggests that under resource‐limited conditions, communities may optimize resource allocation to enhance efficiency (Petchey and Gaston 2002; Mason et al. 2005). By contrast, in the entrance zone—where resources are more plentiful—intensified competitive exclusion favored a few dominant functional groups, resulting in reduced FEve and greater trait inequality, thereby underscoring the combined influence of resource enrichment and competitive pressure on functional structure. Furthermore, as FDiv quantifies the spread of species abundances within functional space, it reflects the degree of ecological niche complementarity (Villeger et al. 2008). The observed decline in FDiv along the increasing light gradient implies a weakening of niche complementarity, likely driven by enhanced trait divergence as species adapt to mitigate interspecific competition. In resource‐rich habitats, traits tend to diverge more strongly, reducing niche overlap and functional redundancy, ultimately leading to lower FDiv values along the photic gradient (Villeger et al. 2008). This trait dispersion highlights the joint regulatory roles of resource availability and competition intensity in shaping community functional architecture. Notably, the community‐weighted mean (CWM) body size of mesostigmatid mites displayed an opposite pattern to the functional diversity metrics, with the smallest sizes recorded in the dark zone. This body size gradient may reflect adaptive strategies among soil microarthropods in response to environmental stress: smaller sizes in resource‐poor settings likely reduce metabolic demands (Gobbi and Fontaneto 2008), while larger sizes in better‐lit, resource‐rich zones may enhance mobility and feeding efficiency, conferring a competitive edge. Together, these trait‐ and diversity‐based patterns provide compelling evidence that cave environmental gradients are key determinants of community assembly processes and functional adaptation.

### 4.2 Co‐Occurrence Networks of Mite Communities and Trophic Interactions Among Soil Mite Groups Across Cave Habitats

Consistent with our second hypothesis, our results show that the complexity of mite co‐occurrence networks increases from the dark zone to the entrance. In this cave ecosystem, predatory mites play a key role in these interaction networks. Along this environmental gradient, rising soil temperatures, more favorable humidity, and increased nutrient availability (e.g., organic matter) collectively promote species richness, leading to more intricate interspecific interactions. The ecological mechanisms behind this phenomenon are well established in soil micro‐food webs. For example, studies have shown that improved environmental conditions stimulate microbial metabolic activity, intensifying competition for limited resources while also fostering mutualistic cooperation; these processes collectively enhance the complexity and connectivity of microbial interaction networks (Morriën et al. 2017). This resource‐driven pattern of network complexification also applies to mite communities. Increased resource availability not only supports higher diversity but also increases the proportion of negative, competition‐dominated interactions within the network. This indicates intensified competition for resources in the relatively rich entrance zone (Long et al. 2025; Guo and Zhang 2025). Conversely, in the resource‐poor dark zone, species often reduce direct competition through niche specialization, resulting in a simpler network structure (Culver and Pipan 2019; Mammola et al. 2020). Ultimately, the gradient in network complexity corroborates the α‐diversity pattern, further confirming that the distinct habitats formed by the cave's light gradient are a key driver of community assembly (Mammola et al. 2020; Souza‐Silva et al. 2021).

Multitrophic network analysis further reveals the central role of predatory mites within the community. The analysis shows that predatory mites not only dominate overall community interactions but also exhibit particularly strong connections with fungivorous mites and primary decomposers. In this study, we identified a total of 18 key taxa, all of which belong to predatory or fungivorous mites, with predatory mites being particularly prominent. This finding corroborates the central role of predators in food webs, who influence the entire community by regulating trophic cascades (Liang et al. 2005). Our findings indicate that predatory mites in caves act as key hubs, maintaining network structure by forming stronger connections with other trophic groups, thereby significantly enhancing overall network complexity and connectivity. Although secondary fungivores (17.69%) and primary decomposers (1.63%) constitute a notable proportion of the community, they exhibit almost no competitive interactions (negative interactions account for only 0.65%). This phenomenon likely stems from the resource allocation characteristics of the cave environment: in extreme habitats like the dark zone, limited resources drive different species to develop highly specialized resource‐use strategies, thus reducing niche overlap within the same trophic level (Gibert and Deharveng 2002; Fišer et al. 2012). In contrast, predators forge tight connections across different trophic groups through extensive trophic cascading effects (positive interactions account for 15.73%), forming a subterranean network structure dominated by predatory links. This structural feature suggests that, along the cave's environmental gradient, the mite community may maintain system stability through a “top‐down” regulatory mechanism. This finding corroborates the conclusions of Parimuchová et al. (2021) from the Ardo vská Cave, who also found that the top predator, the genus Gamasina, triggered trophic cascades by preying on mid‐level consumers like Collembola and small nematodes. The generalist nature of these predators allows them to flexibly respond to resource fluctuations across different environmental zones. This adaptability enables them to act as functional hubs in a resource‐variable environment, thereby solidifying their central position within the interaction network.

### 4.3 Limitations

This study reveals the multidimensional diversity changes of soil mite communities and their trophic groups along a light gradient in caves, and highlights the pivotal role of predatory mites. However, it should be pointed out that this study still has some limitations. First, when using stable isotope datasets to classify mite trophic groups, we faced a universal challenge. Due to the extremely high taxonomic diversity and potential trophic plasticity of soil mites, coupled with the scarcity of isotope data for most species globally, researchers often have to make inferences at higher taxonomic levels such as genus or family. The inherent limitation of this high‐level taxonomic approach is that it may not fully capture fine‐scale trophic niche differentiation at the species level, thereby affecting the precision of the analysis to some extent (Pollierer and Scheu 2021; Maraun et al. 2023). Therefore, caution is required when interpreting the relevant results. Second, the trophic group data referenced in this study are primarily derived from typical terrestrial ecosystems such as forests and grasslands. In contrast, the karst caves focused on in this study have ecological conditions characterized by nutrient poverty and high environmental stability (Engel et al. 2010), which differ from the aforementioned ecosystems. This difference in ecological background means that directly applying existing data to the cave environment may introduce biases in trophic group classification. On the other hand, recent studies have also reported that soil mites exhibit high consistency across different environmental gradients (Lu et al. 2022). Despite some controversy, the application of stable isotope data in this study, compared to traditional laboratory observation methods, still represents an expansion of existing research tools. These preliminary findings also lay an important foundation and point the way for future research that combines molecular biology or more refined stable isotope techniques to further elucidate their precise trophic relationships and community assembly mechanisms.

## 5 Conclusion

We investigated the multidimensional diversity, community structure, and ecological network dynamics of soil mite communities along light gradients in cave habitats. Our findings reveal that, compared to external soil habitats such as cave entrances and farmland, cave‐dwelling soil mites exhibit reduced trophic group complexity, lower diversity, and simpler ecological network structures. Moreover, predatory mites not only represent key taxa within cave ecosystems but also play a central role in mediating trophic interactions among different mite groups. Collectively, these results not only elucidate the multidimensional ecological patterns of soil arthropod communities in extreme environments but also underscore the pivotal role of predatory guilds in sustaining ecosystem stability. These findings offer important theoretical insights into the mechanisms of community assembly, the maintenance of ecological stability, and the conservation of subterranean biodiversity under extreme environmental conditions.

## Author Contributions

**Yan Shen:** conceptualization (lead), data curation (lead), formal analysis (lead), investigation (lead), methodology (lead), software (lead), validation (lead), visualization (lead), writing – original draft (lead), writing – review and editing (lead). **Qiang Wei:** conceptualization (equal), data curation (equal), formal analysis (equal), funding acquisition (lead), investigation (equal), methodology (equal), project administration (lead), software (equal), validation (equal), visualization (equal), writing – original draft (equal), writing – review and editing (equal). **Yuanyuan Zhou:** writing – review and editing (supporting). **Ting Song:** investigation (supporting). **Yihui Liu:** investigation (supporting). **Xiaoxi Lyu:** investigation (supporting). **Hua Xiao:** investigation (supporting). **Hu Chen:** funding acquisition (lead), investigation (supporting), methodology (supporting), project administration (lead), writing – review and editing (supporting).

## Conflicts of Interest

The authors declare no conflicts of interest.

## Supporting information


**Appendix S1:** ece372505‐sup‐0001‐AppendixS1.docx.

## Data Availability

Data are available in Figshare at https://doi.org/10.6084/m9.figshare.30305299.
