# Supplementary material for: Diversity Patterns and Ecological Network Features of Soil Mite Trophic Groups in Karst Cave Ecosystems
Source: Ecol Evol. 2025 Nov 20;15(11):e72505. doi: 10.1002/ece3.72505 (PMC12634052; doi:10.1002/ece3.72505)
Supplement: Supplementary file 1 — Appendix S1: ece372505‐sup‐0001‐AppendixS1.docx. [file ECE3-15-e72505-s001.docx]

**Supplementary Information**

**Title: Diversity patterns and ecological network features of soil mite trophic groups in karst cave ecosystems**

Yan Shen^1^, Qiang Wei^1^, Yuanyuan Zhou^1,2^, Ting Song^1^, Yihui Liu^1^, Xiaoxi Lyu^1^, Hua Xiao^1^, Hu Chen^1^

^1^School of Karst Science, Guizhou Normal University, Guiyang 550001, China

^2^Key Laboratory for Information System of Mountainous Area and Protection of Ecological Environment of Guizhou Province, Guizhou Normal University, Guiyang 550001, China

***Corresponding**: Hu Chen, School of Karst Science, Guizhou Normal University, Guiyang 550001, China. Email: [gy_chenhu@163.com](mailto:gy_chenhu@163.com)

**Supplementary Information: Figures S1-S2 and Tables S1-S11**

**Supplementary Figures:**

**Figure. S1** Map of the study area and photographs of the sampling sites. (p. 3)

**Figure. S2** Variation in soil environmental factors across dark, twilight, and light cave zones. (p. 4)

**Supplementary Tables:**

**Table S1** Location and plant communities of study caves（p. 5）

**Table S2** Morphological characteristics of the cave（p. 6）

**Table S3** Soil Temperature and Moisture in different habitats (Mean ± SD)（p. 7）

**Table S4** Functional traits of soil mites at the genus level（pp. 8-9）

**Table S5** Relative Abundance and Dominance of Soil Mite Genera in Different Habitats（pp. 10-11）

**Table S6** Comparison of relative abundance of the ten most abundant soil mite genera among different habitats（p. 12）

**Table S7** Indicator species of soil mites for different habitats and their indicator values (IndVal > 70%, p < 0.05)（p. 12）

**Table S8** Decomposition of mite community beta diversity in different cave habitats. (p. 13）

**Table S9** Keystone taxa and their trophic groups of soil mite interaction networks in inside the cave and cave entrance habitats（p. 14）

**Table S10** Network topological properties of mite communities in different habitats（p. 15）

**Table S11** Network topological properties of the mite trophic group network in the cave habitat（p. 15）

**
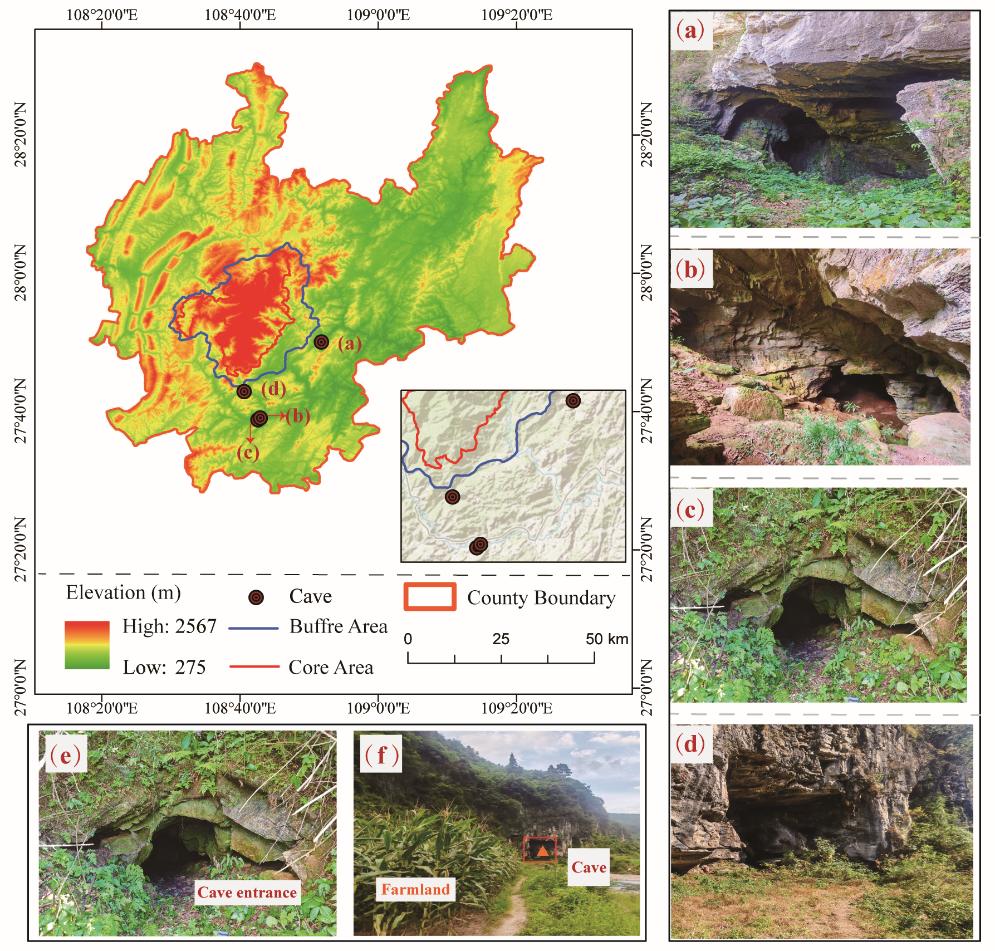
**

**Figure. S1** Map of the study area and photographs of the sampling sites. (a) Shenjia Cave; (b) Longjing Cave; (c) Luzi Cave; (d) Xiong Cave; (e) Cave entrance; (f) Farmland. (Photographed by Yan Shen, July 2024).

**
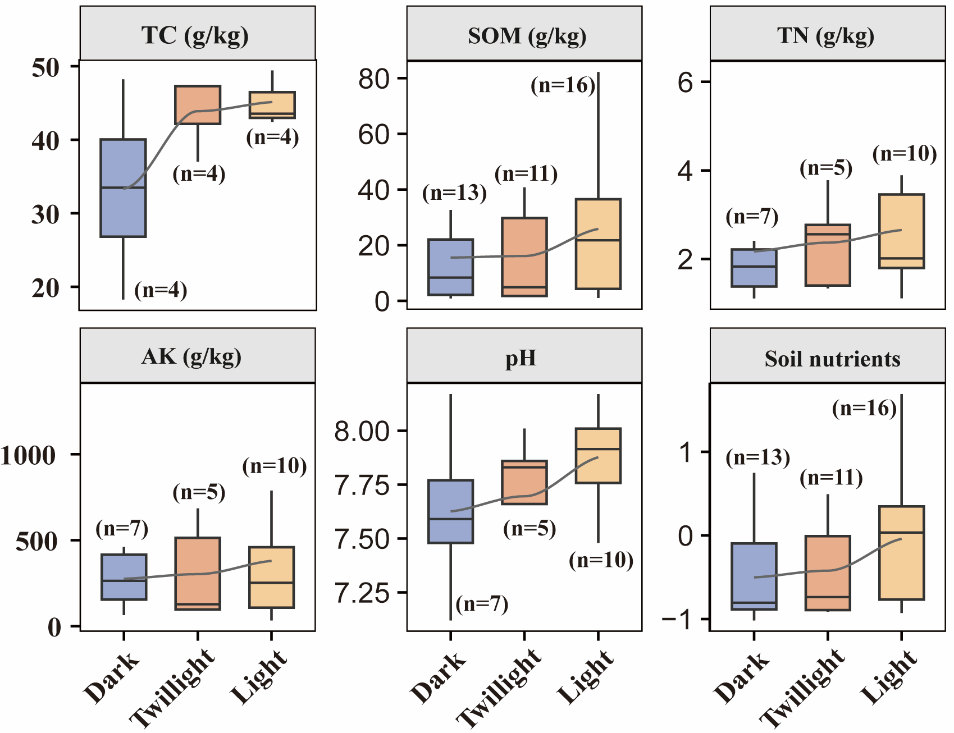
**

**Figure. S2** Variation in soil environmental factors across dark, twilight, and light cave zones. Boxplots show the distribution of total carbon (TC), soil organic matter (SOM), total nitrogen (TN), available potassium (AK), pH, and a comprehensive soil nutrient index within these zones. The nutrient index is the mean of all standardized environmental factors. Data are integrated from this study and 12 published caves (A total of 16 caves; the n in the figure equals the sample size.). Data sources: Li, 2009; Yang et al., 2013; Zhang et al., 2022; Fei et al., 2024.

**Table S1 Location and plant communities of study caves**

| **Study site** | **Longitude and latitude** | **Altitude** | **Orientation** | **Cave plants** |
| --- | --- | --- | --- | --- |
| Shenjia Cave | 108°52′5″E 27°49′56″W | 374.9m | Northwest 336° | *Pteris multifida, Cyrtomium falcatum, Arisaema heterophyllum, Elatostema umbellatum, Nephrolepis cordifolia, Caltha palustris, Clerodendrum bungei, Lonicera japonica, Populusadenopoda, Artemisia dubia, Rosa multiflora, Celastrusangulatus* |
| Luzi Cave | 108°45′0″E 27°39′9″W | 524.4m | Nnortheast 53° | *Senecio scandens, Cyrtomium falcatum, Kalopanax septemlobus, Buddleja officinalis, NBuxus sinica, Artemisia argyi, Clerodendrum bungei, Echinochloa colona, Euonymus fortunei, Artemisia dubia, Fallopia multiflora, Debregeasia orientalis, Celtis sinensis* |
| Longjing Cave | 108°43′18″E 27°38′54″W | 434.9m | West 265° | *Buddleja officinalis, Euonymus fortunei, Arisaema heterophyllum, Solanum nigrum, Nephrolepis cordifolia, Caltha palustris, Clerodendrum bungei, Lonicera japonica, Populus adenopoda, Artemisia dubia, Rosa multiflora, Celastrus angulatus* |
| Xiong Cave | 108°41′12″E 27°43′16″W | 517m | Southeast 148° | *Ilex chinensis, Cyrtomium falcatum, Arisaema heterophyllum, Artemisia argyi, Buddleja officinalis, Caltha palustris, Clerodendrum bungei, Paederia foetida, Populus adenopoda, Artemisia dubia, Rosa multiflora, Fallopia multiflora, Solanum nigrum* |

**Note.** Cave plants are mainly distributed in the entrance and photic zones.

**Table S2 Morphological characteristics of the cave**

| **Study site** | **Cave Length (m)** | **Cave Width (m)** | | | **Cave Height (m)** | | | **Notes** |
| --- | --- | --- | --- | --- | --- | --- | --- | --- |
|  |  | **Range** | **Median** | **Mean±SD** | **Range** | **Median** | **Mean±SD** |  |
| Shenjia Cave | 334.718 | 1.730-14.606 | 4.92 | 4.8±2.5 | 1.5-9.882 | 5.98 | 5.89±3.2 | The cave has a two-tiered structure with thick accumulations of sediment and breakdown piles. Additionally, an underground river flows through the  cave. |
| Luzi Cave | 121.110 | 2.48-10.791 | 8.39 | 7.05±3.7 | 1.554-13.549 | 5.86 | 6.47±3.2 | The cave is rich in speleothems (cave deposits), including secondary carbonate deposits such as stalactites, stalagmites, and flowstones. These features are continuously distributed from the entrance to the deepest parts of the cave. A colony of bats, specially adapted to the environment, inhabits the dark zone. |
| Longjing Cave | 101.584 | 3.009-24.552 | 15.36 | 13.56±7.3 | 5.898-24.417 | 10.01 | 12.53±8.5 | An underground river with outward flow has developed within this cave. The sediment characteristics are relatively homogeneous, dominated by rock pillars and breakdown. During high-water periods, the rising water table can partially flood the cave. |
| Xiong Cave | 90.43 | 2.352-9.778 | 5.38 | 6.54±2.9 | 3.453 | 6.13 | 5.97±4.3 | Compared to the other caves, this cave is relatively small and is located halfway up the mountain. It contains few cave sediments but is inhabited by a large colony of bats. |

**Table S3** **Soil Temperature and Moisture in different habitats (Mean ± SD)**

| **Study site** | **Dark zone** | | **Photic zone** | | **Entrance zone** | | **Farmland zone** | |
| --- | --- | --- | --- | --- | --- | --- | --- | --- |
|  | T(℃) | SM(%) | T(℃) | SM(%) | T(℃) | SM(%) | T(℃) | SM(%) |
| Shenjia Cave | 12.1±0.4 | 88.8±2.4 | 13.2±1.6 | 87.3±3.3 | 15.3±0.5 | 86.7±2.9 | 29.3±0.5 | 83.5±3.3 |
| Luzi Cave | 13.3±0.8 | 87.7±2.8 | 14.7±0.8 | 87.6±1.5 | 16.7±0.8 | 81.2±4.2 | 27.7±0.8 | 82.8±1.7 |
| Longjing Cave | 8.3±0.5 | 87.8±1.7 | - | - | 14.7±1.4 | 82.7±4.6 | 28.8±0.7 | 81.2±3.5 |
| Xiong Cave | 10.3±0.5 | 88.4±1.6 | 13.2±1.4 | 87.6±1.5 | 14.8±1.2 | 84.7±6.4 | 28.5±0.7 | 83.6±3.0 |

Note. T, temperature; SM, soil moisture. Soil temperature and moisture were measured using a SYS-WS soil temperature and moisture meter. (Saiaisi, Jinan, China).

**Table S4 Functional traits of soil mites at the genus level**

| **Family** | **Genus** | **Body length（μm）** | **Body width（μm）** | **FM**  **（μg）** | **n** | **Trophic Group** |
| --- | --- | --- | --- | --- | --- | --- |
| **Oribatida** | |  |  |  |  |  |
| Hypochthoniidae | *Eohypochthonius* | 311.900 | 154.100 | 3.1927 | 10 | pred |
|  | *Hypochthonius* | 150.204 | 252.714 | 16.8241 | 7 | pred |
| Cosmochthoniidae | *Cosmochthonius* | 316.250 | 176.583 | 3.9297 | 12 | fungi |
| Sphaerochthoniidae | *Sphaerochthonius* | 301.000 | 206.000 | 3.6704 | 4 | fungi |
| Haplochthoniidae | *Haplochthonius* | 267.571 | 118.143 | 1.6421 | 7 | fungi |
| Lohmanniidae | *Mixacarus* | 456.667 | 234.667 | 11.5042 | 3 | fungi |
|  | *Papillacarus* | 460.889 | 227.185 | 12.6288 | 33 | fungi |
| Epilohmanniidae | *Epilohmannia* | 400.516 | 165.077 | 6.3924 | 108 | decomp |
| Euphthiracaridae | *Microtritia* | 623.400 | 284.400 | 23.3653 | 5 | fungi |
|  | *Rhysotritia* | 561.875 | 265.813 | 18.2923 | 16 | fungi |
|  | *Acrotritia* | 570.400 | 268.400 | 18.4581 | 5 | fungi |
| Phthiracaridae | *Phthiracarus* | 870.300 | 459.600 | 91.8524 | 10 | fungi |
|  | *Hoplophorella* | 570.500 | 285.500 | 20.3183 | 2 | fungi |
| Camisiidae | *Camisia* | 314.667 | 157.333 | 3.6332 | 3 | lichen |
|  | *Heminothrus* | 367.000 | 172.000 | 4.7284 | 1 | decomp |
| Nothridae | *Nothrus* | 543.182 | 291.636 | 23.9484 | 49 | fungi |
| Nanhermanniidae | *Nanhermannia* | 481.000 | 201.000 | 9.0779 | 1 | fungi |
|  | *Masthermannia* | 480.889 | 202.444 | 9.2607 | 9 | pred |
| Ceratozetidae | *Ceratozetes* | 416.444 | 245.353 | 15.2624 | 18 | pred |
| Punctoribatidae | *Punctoribates* | 458.000 | 325.000 | 17.5679 | 18 | fungi |
| Hermanniellidae | *Hermanniella* | 685.000 | 469.000 | 57.0052 | 1 | decomp |
| Damaeidae | *Epidamaeus* | 285.000 | 149.000 | 4.8304 | 3 | fungi |
|  | *Dyobelba* | 497.556 | 301.333 | 18.2856 | 9 | fungi |
| Eremulidae | *Eremulus* | 397.917 | 255.667 | 9.5565 | 12 | fungi |
| Eremobelbidae | *Eremobelba* | 533.125 | 329.563 | 26.2349 | 16 | fungi |
| Damaeolidae | *Fosseremus* | 217.167 | 124.167 | 1.353 | 6 | fungi |
| Eremaeidae | *Eremaeus* | 405.000 | 242.000 | 9.2695 | 1 | fungi |
| Liacaridae | *Liacarus* | 511.000 | 392.000 | 27.671 | 1 | decomp |
| Ceratoppiidae | *Ceratoppia* | 469.000 | 279.235 | 14.5609 | 17 | decomp |
| Astegistidae | *Cultroribula* | 278.143 | 186.929 | 4.8239 | 21 | fungi |
|  | *Astegistes* | 284.269 | 182.577 | 4.5479 | 27 | fungi |
| Carabodidae | *Carabodes* | 343.000 | 177.000 | 4.4546 | 1 | decomp |
| Suctobelbidae | *Suctobelbella* | 167.000 | 100.000 | 0.63 | 2 | fungi |
| Oppiidae | *Oppia* | 312.000 | 155.500 | 3.3248 | 2 | pred |
|  | *Oppiella* | 251.938 | 132.813 | 1.9092 | 16 | pred |
|  | *Ramusella* | 255.472 | 130.921 | 1.8238 | 127 | pred |
|  | *Microppia* | 156.800 | 82.050 | 0.4574 | 21 | pred |
|  | *Multioppia* | 296.558 | 157.714 | 3.0588 | 96 | fungi |
|  | *Lasiobelba* | 373.667 | 216.333 | 7.3766 | 3 | pred |
|  | *Quadroppia* | 180.500 | 101.000 | 0.7094 | 2 | pred |
| Tectocepheidae | *Tectocepheus* | 275.573 | 165.027 | 2.943 | 111 | decomp |
| Passalozetidae | *Scutovertex* | 228.818 | 123.364 | 1.4679 | 11 | decomp |
| Scheloribatidae | *Scheloribates* | 382.741 | 239.030 | 8.8147 | 211 | fungi |
| Haplozetidae | *Rostrozetes* | 324.000 | 185.333 | 4.7559 | 2 | fungi |
|  | *Xylobates* | 338.667 | 173.333 | 4.3852 | 4 | fungi |
|  | *Peloribates* | 276.000 | 186.667 | 3.4215 | 4 | fungi |
| Protoribatidae | *Protoribates* | 444.632 | 241.849 | 13.6123 | 106 | fungi |
| Galumnidae | *Galumna* | 692.667 | 516.333 | 77.6423 | 6 | fungi |
|  | *Pergalumna* | 579.545 | 425.273 | 41.5808 | 11 | fungi |
|  | *Galumnella* | 313.636 | 219.545 | 5.7421 | 22 | fungi |
| Galumnellidae | *Trichogalumna* | 382.750 | 302.000 | 12.1309 | 4 | fungi |
| Oribatulidae | *Zygoribatula* | 336.635 | 204.923 | 5.5632 | 52 | fungi |
| **Mesostigmata** | |  |  |  |  |  |
| Aceosejidae | *Lasioseius* | 592.176 | 279.667 | 24.543 | 51 | pred |
|  | *Pachyseius* | 757.850 | 382.800 | 49.5561 | 20 | pred |
| Pachylaelapidae | *Gamasholaspis* | 484.808 | 248.962 | 12.9329 | 26 | pred |
|  | *Geolaelaps* | 637.379 | 234.576 | 18.8982 | 66 | pred |
| Parholaspidae | *Asca* | 373.941 | 179.412 | 5.5056 | 20 | pred |
|  | *Gsamasiphis* | 439.792 | 230.208 | 10.9605 | 24 | pred |
|  | *Rhodacarus* | 356.053 | 105.632 | 2.5512 | 19 | pred |
| Hypoaspidae | *Xenozercon* | 380.154 | 223.000 | 7.5135 | 13 | pred |
|  | *Zercon* | 437.818 | 271.818 | 12.9978 | 11 | pred |
|  | *Oplitis* | 579.000 | 484.667 | 46.3576 | 3 | pred |
| Rhodacaridae | *Nenteria* | 465.000 | 340.000 | 21.4566 | 5 | pred |
|  | *Macrocheles* | 1124.304 | 521.652 | 156.7221 | 23 | pred |
|  | *Parholaspius* | 789.727 | 341.000 | 46.639 | 22 | pred |
| Ologammasidae | *Parasitus* | 857.359 | 344.410 | 52.7585 | 39 | pred |
| Zerconidae | *Krantzholaspis* | 853.714 | 332.857 | 57.1226 | 7 | pred |
|  | *Neogamasus* | 725.667 | 316.000 | 43.2111 | 33 | pred |
| Trachyuropodidae | *Amblyseius* | 398.000 | 228.000 | 8.278 | 2 | pred |
| Trematuridae | *Dinychus* | 835.250 | 541.250 | 97.9828 | 8 | pred |
| Macrochelidae | *Cosmolaelaps* | 417.000 | 257.000 | 10.6272 | 1 | pred |
|  | *Cheiroseius* | 527.000 | 290.000 | 18.2917 | 1 | pred |
| Parasitidae | *Glyptholaspis* | 565.500 | 1199.500 | 178.8081 | 2 | pred |
|  | *Pachylaelaps* | 879.000 | 460.000 | 81.0469 | 1 | pred |
| Phytoseiidae | *Epicriopsis* | 296.000 | 205.000 | 4.4512 | 1 | pred |
| Urodinychidae | *Pneumolaelaps* | 601.000 | 232.000 | 16.089 | 2 | pred |
| Ameroseiidae | *Epicrius* | 344.000 | 175.000 | 4.3974 | 1 | pred |
| Epicriidae | *Dendrolaelaps* | 396.857 | 310.571 | 5.3974 | 7 | pred |

**Note.** FM, fresh mass; n, number of individuals measured per genus. The body length and width of soil mites were measured using a VHX-5000 microscope. (Keyence, Osaka, Japan). pred, predators; fungi, fungal feeders; decomp, primary decomposers; lichen, lichen feeders.

**Table S5** **Relative abundance and dominance of soil mite genera in different habitats**

| **Family** | **Genus** | **Dark zone** | **Photic zone** | **Entrance zone** | **Farmland zone** | **Dominance** |
| --- | --- | --- | --- | --- | --- | --- |
| **Oribatida** | |  |  |  |  |  |
| Hypochthoniidae | *Eohypochthonius* | 2 | 0 | 10 | 0 | + |
|  | *Hypochthonius* | 0 | 3 | 2 | 4 | + |
| Cosmochthoniidae | *Cosmochthonius* | 9 | 0 | 3 | 0 | + |
| Sphaerochthoniidae | *Sphaerochthonius* | 0 | 4 | 0 | 0 | + |
| Haplochthoniidae | *Haplochthonius* | 6 | 1 | 3 | 6 | + |
| Lohmanniidae | *Mixacarus* | 0 | 3 | 0 | 0 | + |
|  | *Papillacarus* | 3 | 18 | 15 | 0 | ++ |
| Epilohmanniidae | *Epilohmannia* | 32 | 40 | 112 | 1 | ++ |
| Euphthiracaridae | *Microtritia* | 0 | 24 | 4 | 5 | ++ |
|  | *Rhysotritia* | 0 | 1 | 19 | 15 | ++ |
|  | *Acrotritia* | 5 | 0 | 0 | 0 | + |
| Phthiracaridae | *Phthiracarus* | 0 | 1 | 10 | 1 | + |
|  | *Hoplophorella* | 0 | 0 | 0 | 2 | + |
| Camisiidae | *Camisia* | 0 | 3 | 1 | 0 | + |
|  | *Heminothrus* | 1 | 0 | 1 | 0 | + |
| Nothridae | *Nothrus* | 8 | 7 | 53 | 10 | ++ |
| Nanhermanniidae | *Nanhermannia* | 11 | 0 | 0 | 1 | + |
|  | *Masthermannia* | 0 | 0 | 10 | 0 | + |
| Ceratozetidae | *Ceratozetes* | 0 | 1 | 9 | 9 | + |
| Punctoribatidae | *Punctoribates* | 0 | 1 | 0 | 0 | + |
| Hermanniellidae | *Hermanniella* | 0 | 0 | 1 | 0 | + |
| Damaeidae | *Epidamaeus* | 1 | 2 | 0 | 0 | + |
|  | *Dyobelba* | 0 | 0 | 9 | 0 | + |
| Eremulidae | *Eremulus* | 0 | 0 | 13 | 0 | + |
| Eremobelbidae | *Eremobelba* | 0 | 4 | 1 | 1 | + |
| Damaeolidae | *Fosseremus* | 1 | 0 | 5 | 0 | + |
| Eremaeidae | *Eremaeus* | 0 | 0 | 1 | 0 | + |
| Liacaridae | *Liacarus* | 0 | 0 | 1 | 0 | + |
| Ceratoppiidae | *Ceratoppia* | 0 | 2 | 22 | 0 | + |
| Astegistidae | *Cultroribula* | 1 | 7 | 26 | 1 | ++ |
|  | *Astegistes* | 3 | 0 | 24 | 5 | ++ |
| Carabodidae | *Carabodes* | 1 | 0 | 0 | 0 | + |
| Suctobelbidae | *Suctobelbella* | 3 | 6 | 88 | 1 | ++ |
| Oppiidae | *Oppia* | 3 | 0 | 0 | 0 | + |
|  | *Oppiella* | 7 | 4 | 4 | 0 | + |
|  | *Ramusella* | 26 | 13 | 32 | 23 | ++ |
|  | *Microppia* | 1 | 19 | 1 | 0 | + |
|  | *Multioppia* | 7 | 62 | 14 | 5 | ++ |
|  | *Lasiobelba* | 0 | 5 | 3 | 0 | + |
|  | *Quadroppia* | 0 | 0 | 0 | 1 | + |
| Tectocepheidae | *Tectocepheus* | 2 | 7 | 38 | 162 | ++ |
| Passalozetidae | *Scutovertex* | 1 | 3 | 2 | 10 | + |
| Scheloribatidae | *Scheloribates* | 3 | 20 | 337 | 177 | +++ |
| Haplozetidae | *Rostrozetes* | 0 | 0 | 6 | 2 | + |
|  | *Xylobates* | 3 | 3 | 0 | 0 | + |
|  | *Peloribates* | 0 | 1 | 4 | 0 | + |
| Protoribatidae | *Protoribates* | 1 | 0 | 140 | 36 | ++ |
| Galumnidae | *Galumna* | 1 | 2 | 7 | 0 | + |
|  | *Pergalumna* | 1 | 0 | 17 | 1 | + |
|  | *Galumnella* | 0 | 0 | 4 | 0 | + |
| Galumnellidae | *Trichogalumna* | 1 | 3 | 19 | 1 | + |
| Oribatulidae | *Zygoribatula* | 2 | 0 | 1 | 81 | ++ |
| **Mesostigmata** | |  |  |  |  |  |
| Aceosejidae | *Lasioseius* | 5 | 1 | 53 | 27 | ++ |
|  | *Cheiroseius* | 0 | 1 | 0 | 0 | + |
| Pachylaelapidae | *Pachyseius* | 0 | 0 | 37 | 0 | ++ |
|  | *Pachylaelaps* | 0 | 0 | 6 | 0 | + |
| Parholaspidae | *Gamasholaspis* | 0 | 4 | 13 | 15 | ++ |
|  | *Parholaspius* | 8 | 0 | 7 | 15 | ++ |
|  | *Krantzholaspis* | 0 | 1 | 6 | 1 | + |
| Hypoaspidae | *Geolaelaps* | 9 | 14 | 98 | 35 | ++ |
|  | *Cosmolaelaps* | 0 | 0 | 0 | 1 | + |
|  | *Pneumolaelaps* | 0 | 1 | 0 | 0 | + |
| Rhodacaridae | *Asca* | 3 | 5 | 33 | 2 | ++ |
|  | *Dendrolaelaps* | 1 | 2 | 3 | 1 | + |
|  | *Rhodacarus* | 3 | 3 | 16 | 0 | + |
| Ologammasidae | *Gsamasiphis* | 1 | 2 | 24 | 3 | + |
| Zerconidae | *Xenozercon* | 0 | 0 | 3 | 28 | ++ |
|  | *Zercon* | 0 | 0 | 11 | 0 | + |
| Trachyuropodidae | *Oplitis* | 0 | 0 | 3 | 0 | + |
| Trematuridae | *Nenteria* | 3 | 6 | 4 | 0 | + |
| Macrochelidae | *Macrocheles* | 6 | 12 | 18 | 1 | ++ |
|  | *Glyptholaspis* | 0 | 2 | 0 | 0 | + |
| Parasitidae | *Parasitus* | 0 | 0 | 17 | 32 | ++ |
|  | *Neogamasus* | 0 | 2 | 29 | 20 | ++ |
| Phytoseiidae | *Amblyseius* | 0 | 0 | 1 | 1 | + |
| Urodinychidae | *Dinychus* | 0 | 0 | 0 | 8 | + |
| Ameroseiidae | *Epicriopsis* | 0 | 0 | 0 | 1 | + |
| Epicriidae | *Epicrius* | 1 | 0 | 0 | 0 | + |

**Note.** '+' represents relative abundance of mites ≤1%; '++' represents relative abundance of mites at 1% to 10%; '+++' represents relative abundance of mites ≥10%.

**Table S6 Comparison of relative abundance of the ten most abundant soil mite genera among different habitats**

| **Genus** | **df** | **F-statistic** | **p-value** |
| --- | --- | --- | --- |
| *Scheloribates* | 3 | 29.46 | **0.00** |
| *Tectocepheus* | 3 | 14.31 | **0.00** |
| *Protoribates* | 3 | 55.8 | **0.00** |
| *Epilohmannia* | 3 | 4.039 | **0.03** |
| *Geolaelaps* | 3 | 3.317 | **0.06** |
| *Suctobelbella* | 3 | 14.28 | **0.00** |
| *Lasioseius* | 3 | 3.292 | 0.06 |
| *Zygoribatula* | 3 | 24.42 | **0.00** |
| *Ramusella* | 3 | 0.1944 | 0.89 |
| *Nothrus* | 3 | 0.3272 | 0.80 |

**Table S7 Indicator species of soil mites for different habitats and their indicator values (IndVal > 70%, p < 0.05)**

| **Indicator taxon** | **Dark zone** | **Photic zone** | **Entrance zone** | **Farmland** | **IndVal** | **P value** |
| --- | --- | --- | --- | --- | --- | --- |
| *Eohypochthonius* | 0 | 0 | 1 | 0 | 96.00% | 0.004 |
| *Sphaerochthonius* | 0 | 1 | 0 | 0 | 72.56% | 0.037 |
| *Dyobelba* | 0 | 0 | 1 | 0 | 79.65% | 0.03 |
| *Suctobelbella* | 0 | 0 | 1 | 0 | 70.38% | 0.006 |
| *Microppia* | 0 | 1 | 0 | 0 | 71.43% | 0.012 |
| *Tectocepheus* | 0 | 0 | 0 | 1 | 87.60% | 0.003 |
| *Scutovertex* | 0 | 0 | 0 | 1 | 75.15% | 0.014 |
| *Scheloribates* | 0 | 0 | 1 | 1 | 74.96% | 0.027 |
| *Protoribates* | 0 | 0 | 1 | 0 | 85.07% | 0.001 |
| *Galumna* | 0 | 0 | 1 | 0 | 70.20% | 0.029 |
| *Pergalumna* | 0 | 0 | 1 | 0 | 74.95% | 0.007 |
| *Trichogalumna* | 0 | 0 | 1 | 0 | 75.00% | 0.024 |
| *Zygoribatula* | 0 | 0 | 0 | 1 | 76.37% | 0.004 |
| *Gsamasiphis* | 0 | 0 | 1 | 0 | 83.61% | 0.013 |
| *Dinychus* | 0 | 0 | 0 | 1 | 73.85% | 0.047 |

**Note**. Indicator genera were defined as taxa with an indicator value (IndVal) > 70% and statistical significance (p < 0.05). In the table, “1” denotes a genus that is an indicator for the corresponding habitat.

**Table S8** Decomposition of mite community beta diversity in different cave habitats.

| **Site** | **BD_total_** | **Repl_Total_** | **RichDiff_Total_** | **Repl/BDtotal** | **RichDif/BDtotal** |
| --- | --- | --- | --- | --- | --- |
| Total | 0.3687308 | 0.2074159 | 0.1613149 | 56.25% | 43.75% |
| Inside the cave | 0.3937718 | 0.2530217 | 0.1407502 | 64.26% | 35.74% |
| Dark zone | 0.4068484 | 0.2522073 | 0.1546411 | 61.99% | 38.01% |
| Photic zone | 0.36106398 | 0.29122675 | 0.06983723 | 80.66% | 19.34% |
| Entrance zone | 0.26303778 | 0.20820845 | 0.05482933 | 79.16% | 20.84% |

**Note.** BD_Total_, beta diversity; Repl_Total_, species replacement component; RichDiff_Total_, richness difference component. Repl/BD_Total_, the percentage contribution of the species replacement component to total beta diversity; RichDiff/BD_Total_, the percentage contribution of the richness difference component to total beta diversity.

**Table S9 Keystone taxa and their trophic groups of soil mite interaction networks in inside the cave and cave entrance habitats**

| **Genus** | **Degree** | **Zi** | **Pi** | **Taxa roles** | **Trophic groups** | **Site** |
| --- | --- | --- | --- | --- | --- | --- |
| *Hypochthonius* | 20 | 0.5394 | 0.635 | Connectors | Predators | Inside the cave |
| *Microtritia* | 23 | -0.9439 | 0.6465 | Connectors | Fungal feeders |  |
| *Phthiracarus* | 20 | 0.5394 | 0.635 | Connectors | Fungal feeders |  |
| *Punctoribates* | 20 | 0.5394 | 0.635 | Connectors | Fungal feeders |  |
| *Suctobelbella* | 31 | 0.4273 | 0.6722 | Connectors | Fungal feeders |  |
| *Lasiobelba* | 20 | 0.5394 | 0.635 | Connectors | Predators |  |
| *Trichogalumna* | 18 | -0.9439 | 0.6235 | Connectors | Fungal feeders |  |
| *Cheiroseius* | 20 | 0.5394 | 0.635 | Connectors | Predators |  |
| *Gamasholaspis* | 20 | 0.5394 | 0.635 | Connectors | Predators |  |
| *Krantzholaspis* | 20 | 0.5394 | 0.635 | Connectors | Predators |  |
| *Geolaelaps* | 22 | -2.4271 | 0.7066 | Connectors | Predators |  |
| *Neogamasus* | 20 | 0.5394 | 0.635 | Connectors | Predators |  |
| *Masthermannia* | 14 | -0.9193 | 0.6224 | Connectors | Predators | Cave entrance |
| *Cultroribula* | 14 | -0.9193 | 0.6224 | Connectors | Fungal feeders |  |
| *Protoribates* | 31 | -0.5381 | 0.6535 | Connectors | Fungal feeders |  |
| *Trichogalumna* | 31 | -0.5381 | 0.6535 | Connectors | Fungal feeders |  |
| *Asca* | 16 | -0.5381 | 0.625 | Connectors | Predators |  |
| *Krantzholaspis* | 24 | -2.4837 | 0.6215 | Connectors | Predators |  |

**Note.** All taxa listed are defined as connectors (Zi < 2.5 and Pi ≥ 0.62), which are key taxa possessing high connectivity both within their own modules and among other modules. Inside the cave includes the dark zone and the photic zone.

**Table S10 Network topological properties of mite communities in different habitats**

| **Topological features** | **Dark zone** | **Photic zone** | **Entrance zone** | **Farmland** |
| --- | --- | --- | --- | --- |
| Nodes | 39 | 44 | 60 | 41 |
| Edges | 239 | 508 | 764 | 299 |
| Positive edges | 197 | 338 | 477 | 206 |
| Negative edges | 42 | 170 | 287 | 93 |
| Average degree | 12.25 | 23.09 | 25.46 | 14.58 |
| Average path length | 1.48 | 1.4 | 1.18 | 1.42 |
| Network density | 0.32 | 0.53 | 0.43 | 0.36 |
| Clustering coefficient | 0.83 | 0.79 | 0.71 | 0.74 |
| Modularity | 0.41 | 0.21 | 0.24 | 0.44 |

**Table S11 Network topological properties of the mite trophic group network in the cave habitat**

| **Topological features** | **Predators** | **Fungal feeders** | **Decomposer** |
| --- | --- | --- | --- |
| Nodes | 32 | 32 | 8 |
| Edges | 79 | 54 | 4 |
| Positive edges | 77 | 52 | 4 |
| Negative edges | 2 | 2 | 0 |
| Average degree | 4.93 | 3.375 | 1 |
| Average path length | 1.45 | 2.15 | 0.69 |
| Network density | 0.15 | 0.11 | 0.14 |
| Clustering coefficient | 0.58 | 0.64 | 1 |
| Modularity | 0.39 | 0.53 | 0.37 |

**References for supplementary material：**

1. Fei, Y., Shi, Z., Zhou, Y., Wei, Q., Liu, Y., Shen, Y., & Chen, H. (2024). Distribution pattern and driving factors of mite communities in karst cave ecosystems. Ecology and Evolution, 14(8), e11527.
2. Zhang, S. Q., Du, W. F., Xu, C. X., Hu, B. L., Jiang, X. M., Zhou, F., & Tian, Y. J. (2022). Diversity of cave animal communities and the influence of environmental factors in Xianjia Cave and Yiku Cave, Guizhou. Chinese Journal of Ecology, 41(1), 132–141. (In Chinese)
3. Yang, W. C., Li, D. H., & Xu, C. X. (2013). Analysis on the community structure of cave animals and their relationship with environmental factors in Dayan Cave and Yantou Cave 2, Guangxi. Sichuan Journal of Zoology, 32(3), 442–448. (In Chinese)
4. Li, D. H. (2009). A study on the correlation between some environmental factors and animal community structure in karst caves: A case study of Boduo Cave and Jialiang Cave in Guizhou Province. In Modern Geographical Science and Socioeconomy of Guizhou (pp. 40–51). (In Chinese)
